# The Effect of Hibiscus Sabdariffa on Lipid Profile, Creatinine, and Serum Electrolytes: A Randomized Clinical Trial

**DOI:** 10.5402/2011/976019

**Published:** 2010-11-07

**Authors:** Abbas Mohagheghi, Shirin Maghsoud, Patricia Khashayar, Mohammad Ghazi-Khansari

**Affiliations:** ^1^Department of Cardiology, Shariati Hospital, Tehran University of Medical Sciences, Tehran 14114, Iran; ^2^Medical Student Research Center, School of Medicine, Tehran University of Medical Sciences, Tehran 14114, Iran; ^3^Endocrinology and Metabolism Research Center, Tehran University of Medical Sciences, Kargar st., Tehran 14114, Iran; ^4^Department of Pharmacology, School of Medicine, Tehran University of Medical Sciences, Tehran 14114, Iran

## Abstract

*Background*. Hibiscus Sabdariffa L. (HS), a member of malvaceae family, is a medicinal plant with a worldwide fame. Its effect on reducing serum lipids is mentioned in several studies. The purpose of this study was to assess the efficacy of this plant in reducing the serum's lipids in hypertensive patients. *Materials and Methods*. Ninety hypertensive patients were randomly assigned to receive Hibiscus Sabdariffa (HS) tea or black tea for 15 days. The patients were asked to drink the tea within 20 minutes following its preparation. This process had to be repeated two times, daily. Patient's FBS and lipid profile were collected at the first visit day (day 0) and on the day 30. *Results*. There was no significant differences between pre and post experiment values within the two groups. An upward trend in total cholesterol, HDL, and LDL cholesterol was evident in both groups. The increase in total and HDL cholesterol in both groups relative to their initial values were significant. *Conclusion*. Hibiscus Sabdariffa is probably a safe medicinal plant. No significant harmful changes in cholesterol, triglyceride, BUN, serum creatinine, Na and K levels were observed within 15 days after the discontinuation of the medication.

## 1. Introduction

Hibiscus Sabdariffa L. (HS), a member of malvaceae family, is a medicinal plant with a worldwide fame. It is also known as Roselle (English), I'Oiselle (French), Spanish (Jamaica), Karkade (Arabic), Bissap (Wolof) [1], and Sour Tea (Farsi).

Its flowers contain polyphenolic acid, flavonoids, and anthocyanins [2] with a considerable antioxidant activity [3–5]. There are numerous studies regarding HS components [6–9] and their multipotential activities in treating different diseases, especially those related to the cardiovascular system [2, 10–15].

The effect of HS on laboratory parameters such as fasting blood sugar (FBS), total cholesterol, low-density lipoprotein cholesterol (LDL-C), high-density lipoprotein cholesterol (HDL-C), triglyceride (TG), serum Na, K, creatinine, and blood urea nitrogen (BUN) is controversial. Several studies have reported positive effects while others have reported negative or no significant effect on these parameters specially in animal models [2, 15].

Since HS is believed to be effective in reducing serum lipid concentration, this study was designed to evaluate this effect in hypertensive patients.

## 2. Material and Methods

Ninty essentially hypertensive patients who had been treated for hypertension for at least 3 months were examined. The patients were explained about the study plan and those who volunteered were enrolled. The written informed consent was obtained from all patients. The ethics of this study was confirmed by the ethics committee of the Vice Chancellor for Research of Tehran University of Medical Sciences.

The patients were randomly assigned to receive the tea preparation of Hibiscus Sabdariffa (HS) or black tea for 15 days. The patients' baseline characteristics in HS and BT groups are outlined in Table 1.

Dry calyx of HS plant was obtained from a herbal medicine store and black tea (boxes of 500 mg Ahmad tea) was obtained from a supermarket in Tehran, Iran. Then, we prepared similar boxes containing 500 mg HS or black tea and put instructions for use inside each box. To avoid any misconceptions, the instructions were also described verbally for each patient before he starts using it. Each patient, either in HS or BT group, had to add 15 mg (about two spoons) of the blended tea in two glasses of boiling water and continue boiling it for about 10–15 minutes. The patients were asked to drink the tea within 20 minutes following its preparation. This process had to be repeated two times, daily. The baseline laboratory data were collected at the first visit day (day 0) and included fasting blood sugar (FBS), total cholesterol, low-density lipoprotein cholesterol (LDL-C), high-density lipoprotein cholesterol (HDL-C), triglyceride (TG), serum Na, K, creatinine, and blood urea nitrogen (BUN).

On the day 30, the same laboratory data as day 0 were collected by the same laboratory. The personnel involved in the collection of the outcome data were unaware of the participants' group assignment.

## 3. Statistical Analysis

The data are expressed as mean ± SD. The missing values were excluded from the calculations of the means. The comparisons between medication groups were made with the use of a paired t-test at a specific time. The comparisons between groups were made with the use of an unpaired t-test. All *P*-values are two-sided.

## 4. Results

Out of 90 patients, 4 people (1 BT and 3 HS) refused to refer for the first follow-up visit and 2 people (2 BT) refused to refer after the first follow-up visit due to nonmedical reasons. These people were excluded from the study and are not presented in any result figures. Our study entailed 42 patients in the HS and 42 patients in the BT group. The comparisons of initial fasting blood glucose, lipid profile, blood urea nitrogen (BUN), creatinine (Cr), and serum electrolytes (Na & K) are presented in Table 2.

There were no significant differences between pre- and postexperiment values within the two groups. An upward trend in total cholesterol, HDL, and LDL cholesterol is evident in both groups. The increases in total cholesterol in addition to HDL cholesterol in HS and BT groups relative to their initial values were significant.

## 5. Discussion

The exact effect of HS on lipid profile is unclear. Human and animal studies illustrate different results. It was previously reported that a 10-week administration of HS extract (%0.5 and %1) to cholesterol-fed rabbits resulted in %46–%59 fall in the serum triglyceride and a significant decline in the level of total cholesterol and LDL cholesterol in comparison to the control group [2]. However, the daily oral administration of HS extract to spontaneously hypertensive rats and Wistar Kyoto rats led to no significant changes after 30 days [15]. These discrepancies in results may be due to the duration of the studies, amounts of the administered HS, the number of study population, and other factors.

We observed an increasing trend of plasma lipoproteins in both treatment and BT groups. The magnitude of the increase did not differ significantly between the two groups.

But in each group, the total cholesterol, mainly HDL-C, was increased significantly. Since HDL-C is a protective factor for coronary heart diseases, this feature of HS is quite helpful particularly in hypertensive patients. 

HS and glucose metabolism. HS increased the level of fasting blood glucose, though insignificantly (*P* > .05). A similar result was previously reported in an animal model which experienced an insignificant gradual increase in rat blood sugar until day 60 of the experiment following 30 and 60 days of HS infusion [15]. 

HS and kidney. we also assessed the possible effects of HS on the kidney by BUN and creatinine measurement. No significant changes were seen in these indices before and after HS tea administration. When compared to the BT group, again, no significant changes were found. It means that HS tea administration has no harmful short-term effect on these indices. However, this conclusion needs further and longer studies.

Similar findings were shown in a previous experiment after 10 weeks of continuous administration of HS extract in high cholesterol-fed rabbits [1].

We also measured Na and K to assess any electrolyte disturbance; analyses showed no significant changes in Na or K before and after the HS tea administration and also between the two groups (HS and black tea groups). This result shows that HS tea administration induces no harmful changes in these two important body electrolytes [10].

These findings support the previous findings on the effect of HS on the kidney function as well as electrolytes in rats with two-kidneys-one clip renovascular hypertension [2]. We can say that the short-term administration of HS has no harmful effects on the body water and electrolytes balance. 

It could be concluded that Hibiscus Sabdariffa is probably a safe medicinal plant. No significant harmful changes in cholesterol, triglyceride, BUN, serum creatinine, and Na and K levels were observed within 15 days after the discontinuation of the medication.

## Figures and Tables

**Table 1:** Comparison of patients' baseline characteristics in BT and HS groups.

Characteristic	BT	HS
Age (year)	53 ± 11	50 ± 14
Sex (Female)	15 (36)	23 (55)
FBS	90.7 ± 12	94.5 ± 13
TG	163 ± 53	154.9 ± 59
HDL	45.9 ± 8	44.7 ± 7
LDL	120 ± 42	131.6 ± 29
Total CHOL	201.8 ± 52	207.1 ± 39
BUN	20.0 ± 4.7	22.1 ± 3.6
Creatinine	.9 ± .2	1.0 ± .1
Na	144.6 ± 4	145.1 ± 3
K	4.1 ± .2	4.3 ± .1

Data are presented as mean ± SD.

**Table 2:** Comparison of serum sugar, lipid profile, serum urea, and electrolytes on days 0 and 30.

	BT		HS	
	0	30	0	30
FBS	90.7 ± 12.2	90.6 ± 10.3	94.7 ± 13.2	95 ± 12
TG	164.5 ± 53.3	166.3 ± 52.6	153.5 ± 59	157.5 ± 58
TC	201.8 ± 52.8	204.9 ± 50.6*	206 ± 39	212 ± 37*
HDL-C	45.9 ± 8.7	48.2 ± 8.9*	44.3 ± 8.3	46 ± 7.2*
LDL-C	120.9 ± 42	120.9 ± 40.7	130.8 ± 29	133.2 ± 26
Cr	1.0 ± 0.2	1.1 ± 0.1	1.0 ± 0.2	1.0 ± 0.2
BUN	20.0 ± 4.7	20.3 ± 4.3	22.1 ± 3.6	22.2 ± 2.9
Na	145 ± 4	144 ± 3	145 ± 3	144 ± 2
K	4.1 ± 0.2	4.1 ± 0.2	4.2 ± 0.2	4.2 ± 0.2

FBS: fasting blood sugar; TG: triglyceride; TC: total cholesterol; HDL-C: high density lipoprotein cholesterol; LDL-C: low density lipoprotein cholesterol; Cr: creatinine; BUN: blood urea nitrogen; Na: sodium; K: potassium

*P < .05, Significant difference from day 0 of respected group.
